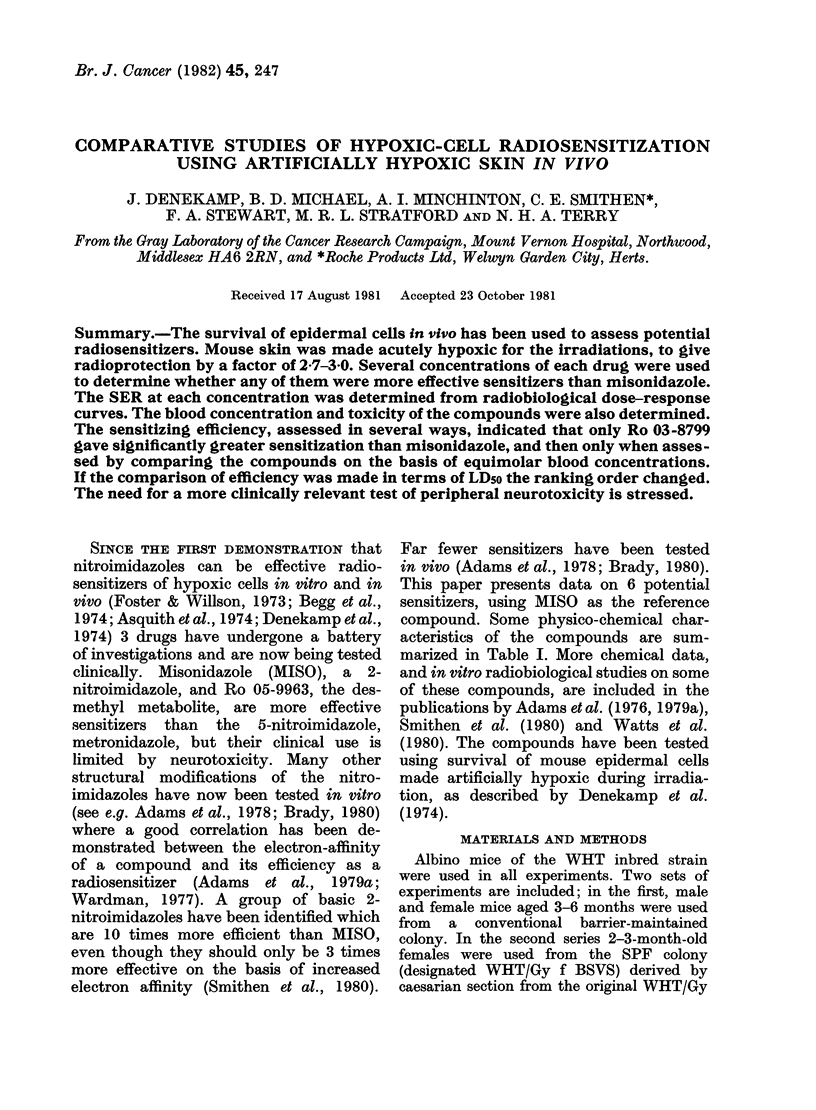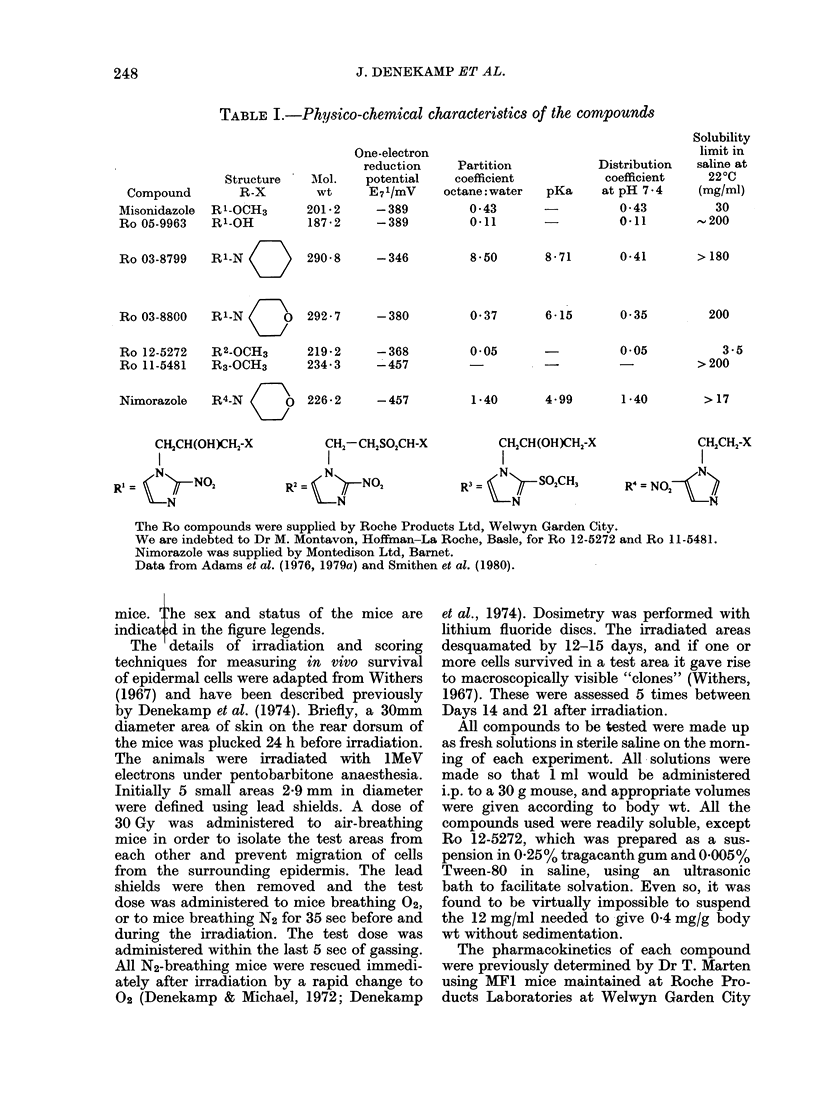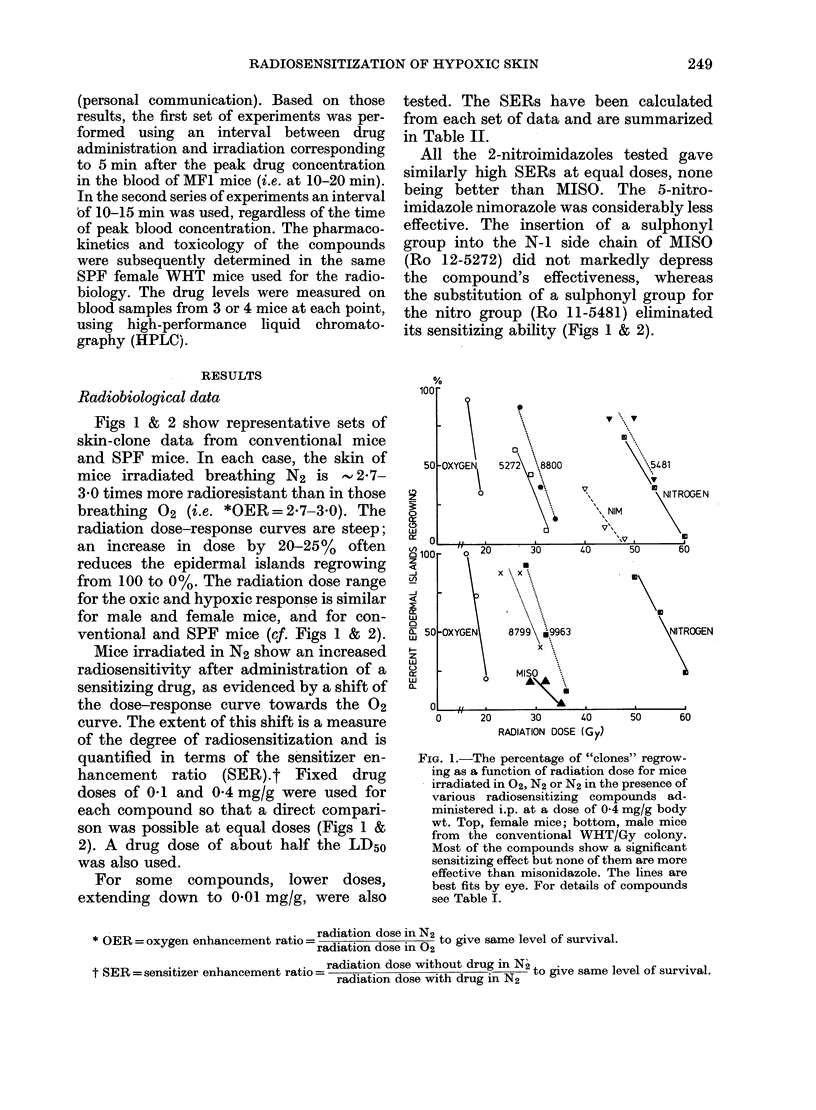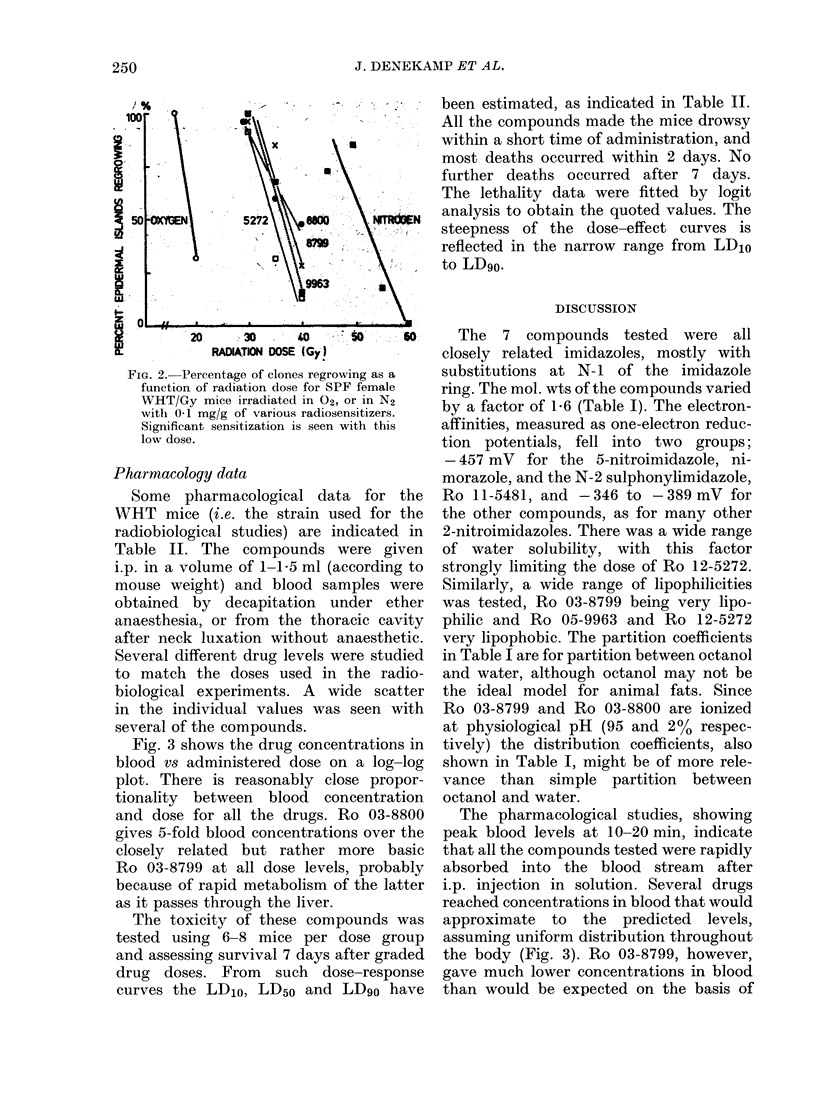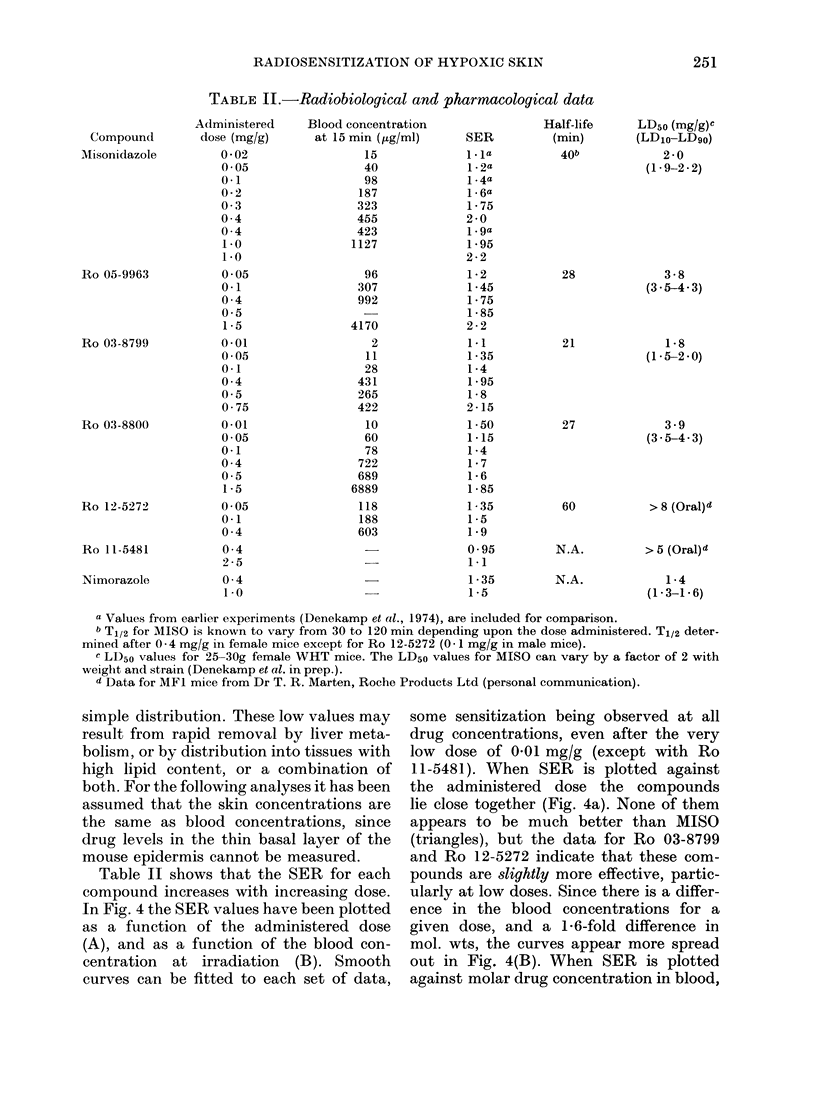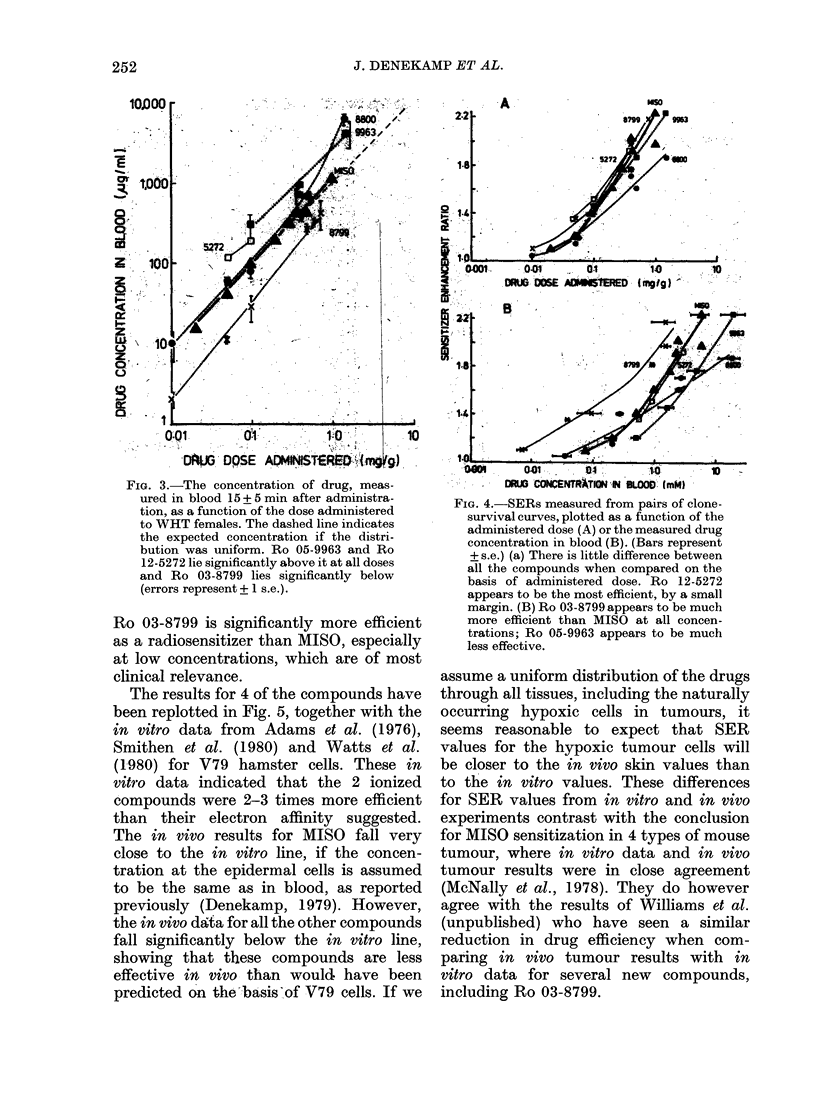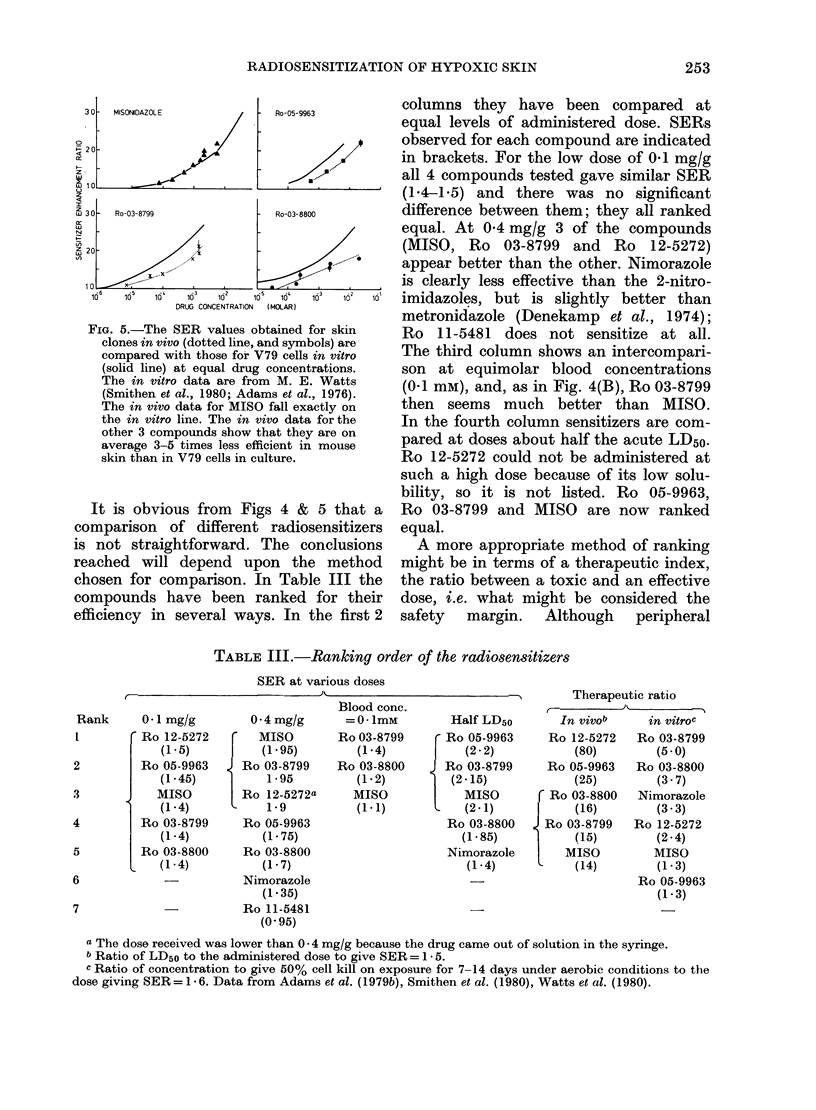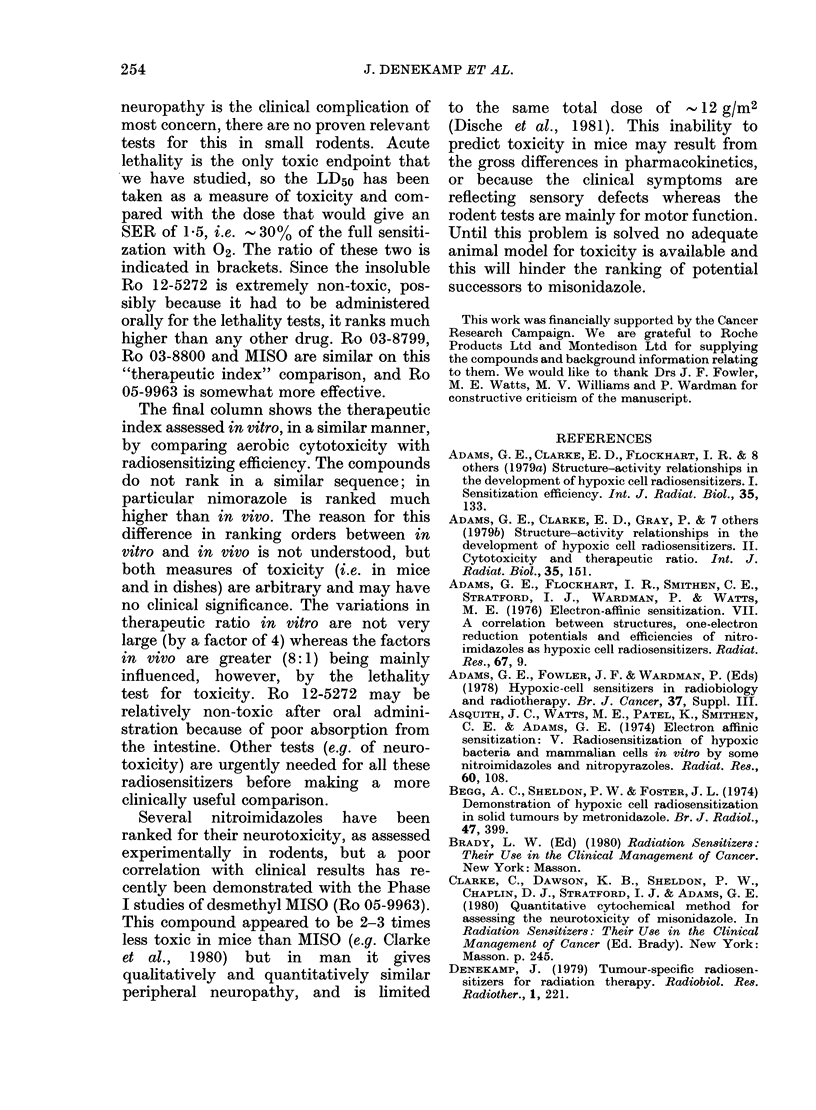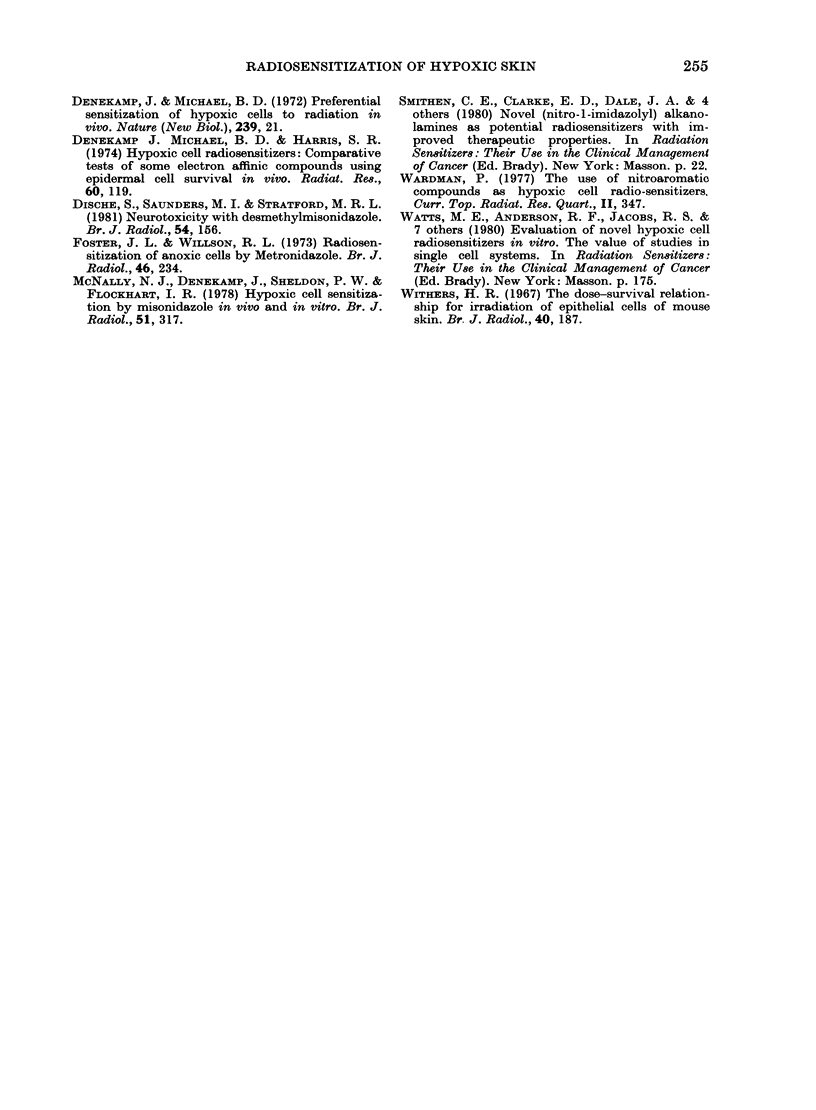# Comparative studies of hypoxic-cell radiosensitization using artificially hypoxic skin in vivo.

**DOI:** 10.1038/bjc.1982.40

**Published:** 1982-02

**Authors:** J. Denekamp, B. D. Michael, A. I. Minchinton, C. E. Smithen, F. A. Stewart, M. R. Stratford, N. H. Terry

## Abstract

The survival of epidermal cells in vivo has been used to assess potential radiosensitizers. Mouse skin was made acutely hypoxic for the irradiations, to give radioprotection by a factor of 2.7-3.0. Several concentrations of each drug were used to determine whether any of them were more effective sensitizers than misonidazole. The SER at each concentration was determined from radiobiological dose-response curves. The blood concentration and toxicity of the compounds were also determined. The sensitizing efficiency, assessed in several ways, indicated that only Ro 03-8799 gave significantly greater sensitization than misonidazole, and then only when assessed by comparing the compounds on the basis of equimolar blood concentrations. If the comparison of efficiency was made in terms of LD50 the ranking order change. The need for a more clinically relevant test of peripheral neurotoxicity is stressed.


*Br. J. Cancer* (1982) **45**, 247

### COMPARATIVE STUDIES OF HYPOXIC-CELL RADIOSENSITIZATION USING ARTIFICIALLY HYPOXIC SKIN *IN VIVO*

J. DENEKAMP, B. D. MICHAEL, A. I. MINCHINTON, C. E. SMITHEN*, F. A. STEWART, M. R. L. STRATFORD AND N. H. A. TERRY

*From the Gray Laboratory of the Cancer Research Campaign, Mount Vernon Hospital, Northwood, Middlesex HA6 2RN, and *Roche Products Ltd, Welwyn Garden City, Herts.*

Received 17 August 1981 Accepted 23 October 1981

**Summary.—The survival of epidermal cells *in vivo* has been used to assess potential radiosensitizers. Mouse skin was made acutely hypoxic for the irradiations, to give radioprotection by a factor of 2·7–3·0. Several concentrations of each drug were used to determine whether any of them were more effective sensitizers than misonidazole. The SER at each concentration was determined from radiobiological dose–response curves. The blood concentration and toxicity of the compounds were also determined. The sensitizing efficiency, assessed in several ways, indicated that only Ro 03-8799 gave significantly greater sensitization than misonidazole, and then only when assessed by comparing the compounds on the basis of equimolar blood concentrations. If the comparison of efficiency was made in terms of $LD_{50}$ the ranking order changed. The need for a more clinically relevant test of peripheral neurotoxicity is stressed.**

SINCE THE FIRST DEMONSTRATION that nitroimidazoles can be effective radiosensitizers of hypoxic cells *in vitro* and *in vivo* (Foster & Willson, 1973; Begg *et al.*, 1974; Asquith *et al.*, 1974; Denekamp *et al.*, 1974) 3 drugs have undergone a battery of investigations and are now being tested clinically. Misonidazole (MISO), a 2-nitroimidazole, and Ro 05-9963, the desmethyl metabolite, are more effective sensitizers than the 5-nitroimidazole, metronidazole, but their clinical use is limited by neurotoxicity. Many other structural modifications of the nitroimidazoles have now been tested *in vitro* (see *e.g.* Adams *et al.*, 1978; Brady, 1980) where a good correlation has been demonstrated between the electron-affinity of a compound and its efficiency as a radiosensitizer (Adams *et al.*, 1979*a*; Wardman, 1977). A group of basic 2-nitroimidazoles have been identified which are 10 times more efficient than MISO, even though they should only be 3 times more effective on the basis of increased electron affinity (Smithen *et al.*, 1980). Far fewer sensitizers have been tested *in vivo* (Adams *et al.*, 1978; Brady, 1980). This paper presents data on 6 potential sensitizers, using MISO as the reference compound. Some physico-chemical characteristics of the compounds are summarized in Table I. More chemical data, and *in vitro* radiobiological studies on some of these compounds, are included in the publications by Adams *et al.* (1976, 1979a), Smithen *et al.* (1980) and Watts *et al.* (1980). The compounds have been tested using survival of mouse epidermal cells made artificially hypoxic during irradiation, as described by Denekamp *et al.* (1974).

### MATERIALS AND METHODS

Albino mice of the WHT inbred strain were used in all experiments. Two sets of experiments are included; in the first, male and female mice aged 3–6 months were used from a conventional barrier-maintained colony. In the second series 2–3-month-old females were used from the SPF colony (designated WHT/Gy f BSVS) derived by caesarian section from the original WHT/Gy

TABLE I.—*Physico-chemical characteristics of the compounds*

| Compound | Structure R-X | Mol. wt | One-electron reduction potential $E_7^1$/mV | Partition coefficient octane:water | pKa | Distribution coefficient at pH 7·4 | Solubility limit in saline at 22°C (mg/ml) |
|---|---|---|---|---|---|---|---|
| Misonidazole | $R^1$-$OCH_3$ | 201·2 | −389 | 0·43 | — | 0·43 | 30 |
| Ro 05-9963 | $R^1$-OH | 187·2 | −389 | 0·11 | — | 0·11 | ~200 |
| Ro 03-8799 | $R^1$-N  | 290·8 | −346 | 8·50 | 8·71 | 0·41 | >180 |
| Ro 03-8800 | $R^1$-N  | 292·7 | −380 | 0·37 | 6·15 | 0·35 | 200 |
| Ro 12-5272 | $R^2$-$OCH_3$ | 219·2 | −368 | 0·05 | — | 0·05 | 3·5 |
| Ro 11-5481 | $R_3$-$OCH_3$ | 234·3 | −457 | — | — | — | >200 |
| Nimorazole | $R^4$-N  | 226·2 | −457 | 1·40 | 4·99 | 1·40 | >17 |

The Ro compounds were supplied by Roche Products Ltd, Welwyn Garden City.
We are indebted to Dr M. Montavon, Hoffman–La Roche, Basle, for Ro 12-5272 and Ro 11-5481.
Nimorazole was supplied by Montedison Ltd, Barnet.
Data from Adams *et al.* (1976, 1979*a*) and Smithen *et al.* (1980).

mice. The sex and status of the mice are indicated in the figure legends.

The details of irradiation and scoring techniques for measuring *in vivo* survival of epidermal cells were adapted from Withers (1967) and have been described previously by Denekamp *et al.* (1974). Briefly, a 30mm diameter area of skin on the rear dorsum of the mice was plucked 24 h before irradiation. The animals were irradiated with 1MeV electrons under pentobarbitone anaesthesia. Initially 5 small areas 2·9 mm in diameter were defined using lead shields. A dose of 30 Gy was administered to air-breathing mice in order to isolate the test areas from each other and prevent migration of cells from the surrounding epidermis. The lead shields were then removed and the test dose was administered to mice breathing $O_2$, or to mice breathing $N_2$ for 35 sec before and during the irradiation. The test dose was administered within the last 5 sec of gassing. All $N_2$-breathing mice were rescued immediately after irradiation by a rapid change to $O_2$ (Denekamp & Michael, 1972; Denekamp *et al.*, 1974). Dosimetry was performed with lithium fluoride discs. The irradiated areas desquamated by 12–15 days, and if one or more cells survived in a test area it gave rise to macroscopically visible "clones" (Withers, 1967). These were assessed 5 times between Days 14 and 21 after irradiation.

All compounds to be tested were made up as fresh solutions in sterile saline on the morning of each experiment. All solutions were made so that 1 ml would be administered i.p. to a 30 g mouse, and appropriate volumes were given according to body wt. All the compounds used were readily soluble, except Ro 12-5272, which was prepared as a suspension in 0·25% tragacanth gum and 0·005% Tween-80 in saline, using an ultrasonic bath to facilitate solvation. Even so, it was found to be virtually impossible to suspend the 12 mg/ml needed to give 0·4 mg/g body wt without sedimentation.

The pharmacokinetics of each compound were previously determined by Dr T. Marten using MF1 mice maintained at Roche Products Laboratories at Welwyn Garden City

(personal communication). Based on those results, the first set of experiments was performed using an interval between drug administration and irradiation corresponding to 5 min after the peak drug concentration in the blood of MF1 mice (*i.e.* at 10–20 min). In the second series of experiments an interval of 10–15 min was used, regardless of the time of peak blood concentration. The pharmacokinetics and toxicology of the compounds were subsequently determined in the same SPF female WHT mice used for the radiobiology. The drug levels were measured on blood samples from 3 or 4 mice at each point, using high-performance liquid chromatography (HPLC).

### RESULTS

### *Radiobiological data*

Figs 1 & 2 show representative sets of skin-clone data from conventional mice and SPF mice. In each case, the skin of mice irradiated breathing $N_2$ is ~2·7–3·0 times more radioresistant than in those breathing $O_2$ (*i.e.* *OER = 2·7–3·0). The radiation dose–response curves are steep; an increase in dose by 20–25% often reduces the epidermal islands regrowing from 100 to 0%. The radiation dose range for the oxic and hypoxic response is similar for male and female mice, and for conventional and SPF mice (*cf.* Figs 1 & 2).

Mice irradiated in $N_2$ show an increased radiosensitivity after administration of a sensitizing drug, as evidenced by a shift of the dose–response curve towards the $O_2$ curve. The extent of this shift is a measure of the degree of radiosensitization and is quantified in terms of the sensitizer enhancement ratio (SER).† Fixed drug doses of 0·1 and 0·4 mg/g were used for each compound so that a direct comparison was possible at equal doses (Figs 1 & 2). A drug dose of about half the $LD_{50}$ was also used.

For some compounds, lower doses, extending down to 0·01 mg/g, were also tested. The SERs have been calculated from each set of data and are summarized in Table II.

All the 2-nitroimidazoles tested gave similarly high SERs at equal doses, none being better than MISO. The 5-nitroimidazole nimorazole was considerably less effective. The insertion of a sulphonyl group into the N-1 side chain of MISO (Ro 12-5272) did not markedly depress the compound's effectiveness, whereas the substitution of a sulphonyl group for the nitro group (Ro 11-5481) eliminated its sensitizing ability (Figs 1 & 2).

FIG. 1.—The percentage of "clones" regrowing as a function of radiation dose for mice irradiated in $O_2$, $N_2$ or $N_2$ in the presence of various radiosensitizing compounds administered i.p. at a dose of 0·4 mg/g body wt. Top, female mice; bottom, male mice from the conventional WHT/Gy colony. Most of the compounds show a significant sensitizing effect but none of them are more effective than misonidazole. The lines are best fits by eye. For details of compounds see Table I.

$$* \text{ OER} = \text{oxygen enhancement ratio} = \frac{\text{radiation dose in } N_2}{\text{radiation dose in } O_2} \text{ to give same level of survival.}$$

$$\dagger \text{ SER} = \text{sensitizer enhancement ratio} = \frac{\text{radiation dose without drug in } N_2}{\text{radiation dose with drug in } N_2} \text{ to give same level of survival.}$$

FIG. 2.—Percentage of clones regrowing as a function of radiation dose for SPF female WHT/Gy mice irradiated in $O_2$, or in $N_2$ with 0·1 mg/g of various radiosensitizers. Significant sensitization is seen with this low dose.

*Pharmacology data*

Some pharmacological data for the WHT mice (*i.e.* the strain used for the radiobiological studies) are indicated in Table II. The compounds were given i.p. in a volume of 1–1·5 ml (according to mouse weight) and blood samples were obtained by decapitation under ether anaesthesia, or from the thoracic cavity after neck luxation without anaesthetic. Several different drug levels were studied to match the doses used in the radiobiological experiments. A wide scatter in the individual values was seen with several of the compounds.

Fig. 3 shows the drug concentrations in blood *vs* administered dose on a log–log plot. There is reasonably close proportionality between blood concentration and dose for all the drugs. Ro 03-8800 gives 5-fold blood concentrations over the closely related but rather more basic Ro 03-8799 at all dose levels, probably because of rapid metabolism of the latter as it passes through the liver.

The toxicity of these compounds was tested using 6–8 mice per dose group and assessing survival 7 days after graded drug doses. From such dose–response curves the $LD_{10}$, $LD_{50}$ and $LD_{90}$ have been estimated, as indicated in Table II. All the compounds made the mice drowsy within a short time of administration, and most deaths occurred within 2 days. No further deaths occurred after 7 days. The lethality data were fitted by logit analysis to obtain the quoted values. The steepness of the dose–effect curves is reflected in the narrow range from $LD_{10}$ to $LD_{90}$.

### DISCUSSION

The 7 compounds tested were all closely related imidazoles, mostly with substitutions at N-1 of the imidazole ring. The mol. wts of the compounds varied by a factor of 1·6 (Table I). The electron-affinities, measured as one-electron reduction potentials, fell into two groups; −457 mV for the 5-nitroimidazole, nimorazole, and the N-2 sulphonylimidazole, Ro 11-5481, and −346 to −389 mV for the other compounds, as for many other 2-nitroimidazoles. There was a wide range of water solubility, with this factor strongly limiting the dose of Ro 12-5272. Similarly, a wide range of lipophilicities was tested, Ro 03-8799 being very lipophilic and Ro 05-9963 and Ro 12-5272 very lipophobic. The partition coefficients in Table I are for partition between octanol and water, although octanol may not be the ideal model for animal fats. Since Ro 03-8799 and Ro 03-8800 are ionized at physiological pH (95 and 2% respectively) the distribution coefficients, also shown in Table I, might be of more relevance than simple partition between octanol and water.

The pharmacological studies, showing peak blood levels at 10–20 min, indicate that all the compounds tested were rapidly absorbed into the blood stream after i.p. injection in solution. Several drugs reached concentrations in blood that would approximate to the predicted levels, assuming uniform distribution throughout the body (Fig. 3). Ro 03-8799, however, gave much lower concentrations in blood than would be expected on the basis of

TABLE II.—*Radiobiological and pharmacological data*

| Compound | Administered dose (mg/g) | Blood concentration at 15 min (μg/ml) | SER | Half-life (min) | $LD_{50}$ (mg/g)[c] ($LD_{10}$–$LD_{90}$) |
|---|---|---|---|---|---|
| Misonidazole | 0·02 | 15 | 1·1[a] | 40[b] | 2·0 |
| | 0·05 | 40 | 1·2[a] | | (1·9–2·2) |
| | 0·1 | 98 | 1·4[a] | | |
| | 0·2 | 187 | 1·6[a] | | |
| | 0·3 | 323 | 1·75 | | |
| | 0·4 | 455 | 2·0 | | |
| | 0·4 | 423 | 1·9[a] | | |
| | 1·0 | 1127 | 1·95 | | |
| | 1·0 | | 2·2 | | |
| Ro 05-9963 | 0·05 | 96 | 1·2 | 28 | 3·8 |
| | 0·1 | 307 | 1·45 | | (3·5–4·3) |
| | 0·4 | 992 | 1·75 | | |
| | 0·5 | — | 1·85 | | |
| | 1·5 | 4170 | 2·2 | | |
| Ro 03-8799 | 0·01 | 2 | 1·1 | 21 | 1·8 |
| | 0·05 | 11 | 1·35 | | (1·5–2·0) |
| | 0·1 | 28 | 1·4 | | |
| | 0·4 | 431 | 1·95 | | |
| | 0·5 | 265 | 1·8 | | |
| | 0·75 | 422 | 2·15 | | |
| Ro 03-8800 | 0·01 | 10 | 1·50 | 27 | 3·9 |
| | 0·05 | 60 | 1·15 | | (3·5–4·3) |
| | 0·1 | 78 | 1·4 | | |
| | 0·4 | 722 | 1·7 | | |
| | 0·5 | 689 | 1·6 | | |
| | 1·5 | 6889 | 1·85 | | |
| Ro 12-5272 | 0·05 | 118 | 1·35 | 60 | > 8 (Oral)[d] |
| | 0·1 | 188 | 1·5 | | |
| | 0·4 | 603 | 1·9 | | |
| Ro 11-5481 | 0·4 | — | 0·95 | N.A. | > 5 (Oral)[d] |
| | 2·5 | — | 1·1 | | |
| Nimorazole | 0·4 | — | 1·35 | N.A. | 1·4 |
| | 1·0 | — | 1·5 | | (1·3–1·6) |

[a] Values from earlier experiments (Denekamp *et al.*, 1974), are included for comparison.
[b] $T_{1/2}$ for MISO is known to vary from 30 to 120 min depending upon the dose administered. $T_{1/2}$ determined after 0·4 mg/g in female mice except for Ro 12-5272 (0·1 mg/g in male mice).
[c] $LD_{50}$ values for 25–30g female WHT mice. The $LD_{50}$ values for MISO can vary by a factor of 2 with weight and strain (Denekamp *et al.* in prep.).
[d] Data for MF1 mice from Dr T. R. Marten, Roche Products Ltd (personal communication).

simple distribution. These low values may result from rapid removal by liver metabolism, or by distribution into tissues with high lipid content, or a combination of both. For the following analyses it has been assumed that the skin concentrations are the same as blood concentrations, since drug levels in the thin basal layer of the mouse epidermis cannot be measured.

Table II shows that the SER for each compound increases with increasing dose. In Fig. 4 the SER values have been plotted as a function of the administered dose (A), and as a function of the blood concentration at irradiation (B). Smooth curves can be fitted to each set of data, some sensitization being observed at all drug concentrations, even after the very low dose of 0·01 mg/g (except with Ro 11-5481). When SER is plotted against the administered dose the compounds lie close together (Fig. 4a). None of them appears to be much better than MISO (triangles), but the data for Ro 03-8799 and Ro 12-5272 indicate that these compounds are *slightly* more effective, particularly at low doses. Since there is a difference in the blood concentrations for a given dose, and a 1·6-fold difference in mol. wts, the curves appear more spread out in Fig. 4(B). When SER is plotted against molar drug concentration in blood,

FIG. 3.—The concentration of drug, measured in blood 15 ± 5 min after administration, as a function of the dose administered to WHT females. The dashed line indicates the expected concentration if the distribution was uniform. Ro 05-9963 and Ro 12-5272 lie significantly above it at all doses and Ro 03-8799 lies significantly below (errors represent ± 1 s.e.).

FIG. 4.—SERs measured from pairs of clone-survival curves, plotted as a function of the administered dose (A) or the measured drug concentration in blood (B). (Bars represent ± s.e.) (a) There is little difference between all the compounds when compared on the basis of administered dose. Ro 12-5272 appears to be the most efficient, by a small margin. (B) Ro 03-8799 appears to be much more efficient than MISO at all concentrations; Ro 05-9963 appears to be much less effective.

Ro 03-8799 is significantly more efficient as a radiosensitizer than MISO, especially at low concentrations, which are of most clinical relevance.

The results for 4 of the compounds have been replotted in Fig. 5, together with the *in vitro* data from Adams *et al.* (1976), Smithen *et al.* (1980) and Watts *et al.* (1980) for V79 hamster cells. These *in vitro* data indicated that the 2 ionized compounds were 2–3 times more efficient than their electron affinity suggested. The *in vivo* results for MISO fall very close to the *in vitro* line, if the concentration at the epidermal cells is assumed to be the same as in blood, as reported previously (Denekamp, 1979). However, the *in vivo* data for all the other compounds fall significantly below the *in vitro* line, showing that these compounds are less effective *in vivo* than would have been predicted on the basis of V79 cells. If we assume a uniform distribution of the drugs through all tissues, including the naturally occurring hypoxic cells in tumours, it seems reasonable to expect that SER values for the hypoxic tumour cells will be closer to the *in vivo* skin values than to the *in vitro* values. These differences for SER values from *in vitro* and *in vivo* experiments contrast with the conclusion for MISO sensitization in 4 types of mouse tumour, where *in vitro* data and *in vivo* tumour results were in close agreement (McNally *et al.*, 1978). They do however agree with the results of Williams *et al.* (unpublished) who have seen a similar reduction in drug efficiency when comparing *in vivo* tumour results with *in vitro* data for several new compounds, including Ro 03-8799.

FIG. 5.—The SER values obtained for skin clones *in vivo* (dotted line, and symbols) are compared with those for V79 cells *in vitro* (solid line) at equal drug concentrations. The *in vitro* data are from M. E. Watts (Smithen *et al.*, 1980; Adams *et al.*, 1976). The *in vivo* data for MISO fall exactly on the *in vitro* line. The *in vivo* data for the other 3 compounds show that they are on average 3–5 times less efficient in mouse skin than in V79 cells in culture.

It is obvious from Figs 4 & 5 that a comparison of different radiosensitizers is not straightforward. The conclusions reached will depend upon the method chosen for comparison. In Table III the compounds have been ranked for their efficiency in several ways. In the first 2 columns they have been compared at equal levels of administered dose. SERs observed for each compound are indicated in brackets. For the low dose of 0·1 mg/g all 4 compounds tested gave similar SER (1·4–1·5) and there was no significant difference between them; they all ranked equal. At 0·4 mg/g 3 of the compounds (MISO, Ro 03-8799 and Ro 12-5272) appear better than the other. Nimorazole is clearly less effective than the 2-nitroimidazoles, but is slightly better than metronidazole (Denekamp *et al.*, 1974); Ro 11-5481 does not sensitize at all. The third column shows an intercomparison at equimolar blood concentrations (0·1 mM), and, as in Fig. 4(B), Ro 03-8799 then seems much better than MISO. In the fourth column sensitizers are compared at doses about half the acute $LD_{50}$. Ro 12-5272 could not be administered at such a high dose because of its low solubility, so it is not listed. Ro 05-9963, Ro 03-8799 and MISO are now ranked equal.

A more appropriate method of ranking might be in terms of a therapeutic index, the ratio between a toxic and an effective dose, *i.e.* what might be considered the safety margin. Although peripheral

TABLE III.—*Ranking order of the radiosensitizers*

| | SER at various doses | | | | Therapeutic ratio | |
|---|---|---|---|---|---|---|
| Rank | 0·1 mg/g | 0·4 mg/g | Blood conc. =0·1mM | Half $LD_{50}$ | *In vivo*[b] | *in vitro*[c] |
| 1 | Ro 12-5272 (1·5) | MISO (1·95) | Ro 03-8799 (1·4) | Ro 05-9963 (2·2) | Ro 12-5272 (80) | Ro 03-8799 (5·0) |
| 2 | Ro 05-9963 (1·45) | Ro 03-8799 1·95 | Ro 03-8800 (1·2) | Ro 03-8799 (2·15) | Ro 05-9963 (25) | Ro 03-8800 (3·7) |
| 3 | MISO (1·4) | Ro 12-5272[a] 1·9 | MISO (1·1) | MISO (2·1) | Ro 03-8800 (16) | Nimorazole (3·3) |
| 4 | Ro 03-8799 (1·4) | Ro 05-9963 (1·75) | | Ro 03-8800 (1·85) | Ro 03-8799 (15) | Ro 12-5272 (2·4) |
| 5 | Ro 03-8800 (1·4) | Ro 03-8800 (1·7) | | Nimorazole (1·4) | MISO (14) | MISO (1·3) |
| 6 | — | Nimorazole (1·35) | | — | | Ro 05-9963 (1·3) |
| 7 | — | Ro 11-5481 (0·95) | | — | | — |

Ranks 1–5 at 0·1 mg/g are bracketed together; ranks 1–3 at 0·4 mg/g are bracketed together; ranks 1–3 at Half $LD_{50}$ are bracketed together; ranks 3–5 of the *in vivo* therapeutic ratio are bracketed together.

[a] The dose received was lower than 0·4 mg/g because the drug came out of solution in the syringe.
[b] Ratio of $LD_{50}$ to the administered dose to give SER = 1·5.
[c] Ratio of concentration to give 50% cell kill on exposure for 7–14 days under aerobic conditions to the dose giving SER = 1·6. Data from Adams *et al.* (1979*b*), Smithen *et al.* (1980), Watts *et al.* (1980).

neuropathy is the clinical complication of most concern, there are no proven relevant tests for this in small rodents. Acute lethality is the only toxic endpoint that we have studied, so the $LD_{50}$ has been taken as a measure of toxicity and compared with the dose that would give an SER of 1·5, *i.e.* ~30% of the full sensitization with $O_2$. The ratio of these two is indicated in brackets. Since the insoluble Ro 12-5272 is extremely non-toxic, possibly because it had to be administered orally for the lethality tests, it ranks much higher than any other drug. Ro 03-8799, Ro 03-8800 and MISO are similar on this "therapeutic index" comparison, and Ro 05-9963 is somewhat more effective.

The final column shows the therapeutic index assessed *in vitro*, in a similar manner, by comparing aerobic cytotoxicity with radiosensitizing efficiency. The compounds do not rank in a similar sequence; in particular nimorazole is ranked much higher than *in vivo*. The reason for this difference in ranking orders between *in vitro* and *in vivo* is not understood, but both measures of toxicity (*i.e.* in mice and in dishes) are arbitrary and may have no clinical significance. The variations in therapeutic ratio *in vitro* are not very large (by a factor of 4) whereas the factors *in vivo* are greater (8:1) being mainly influenced, however, by the lethality test for toxicity. Ro 12-5272 may be relatively non-toxic after oral administration because of poor absorption from the intestine. Other tests (*e.g.* of neurotoxicity) are urgently needed for all these radiosensitizers before making a more clinically useful comparison.

Several nitroimidazoles have been ranked for their neurotoxicity, as assessed experimentally in rodents, but a poor correlation with clinical results has recently been demonstrated with the Phase I studies of desmethyl MISO (Ro 05-9963). This compound appeared to be 2–3 times less toxic in mice than MISO (*e.g.* Clarke *et al.*, 1980) but in man it gives qualitatively and quantitatively similar peripheral neuropathy, and is limited to the same total dose of ~12 g/m$^2$ (Dische *et al.*, 1981). This inability to predict toxicity in mice may result from the gross differences in pharmacokinetics, or because the clinical symptoms are reflecting sensory defects whereas the rodent tests are mainly for motor function. Until this problem is solved no adequate animal model for toxicity is available and this will hinder the ranking of potential successors to misonidazole.

This work was financially supported by the Cancer Research Campaign. We are grateful to Roche Products Ltd and Montedison Ltd for supplying the compounds and background information relating to them. We would like to thank Drs J. F. Fowler, M. E. Watts, M. V. Williams and P. Wardman for constructive criticism of the manuscript.

### REFERENCES

Adams, G. E., Clarke, E. D., Flockhart, I. R. & 8 others (1979*a*) Structure–activity relationships in the development of hypoxic cell radiosensitizers. I. Sensitization efficiency. *Int. J. Radiat. Biol.*, **35**, 133.

Adams, G. E., Clarke, E. D., Gray, P. & 7 others (1979*b*) Structure–activity relationships in the development of hypoxic cell radiosensitizers. II. Cytotoxicity and therapeutic ratio. *Int. J. Radiat. Biol.*, **35**, 151.

Adams, G. E., Flockhart, I. R., Smithen, C. E., Stratford, I. J., Wardman, P. & Watts, M. E. (1976) Electron-affinic sensitization. VII. A correlation between structures, one-electron reduction potentials and efficiencies of nitroimidazoles as hypoxic cell radiosensitizers. *Radiat. Res.*, **67**, 9.

Adams, G. E., Fowler, J. F. & Wardman, P. (Eds) (1978) Hypoxic-cell sensitizers in radiobiology and radiotherapy. *Br. J. Cancer*, **37**, Suppl. III.

Asquith, J. C., Watts, M. E., Patel, K., Smithen, C. E. & Adams, G. E. (1974) Electron affinic sensitization: V. Radiosensitization of hypoxic bacteria and mammalian cells *in vitro* by some nitroimidazoles and nitropyrazoles. *Radiat. Res.*, **60**, 108.

Begg, A. C., Sheldon, P. W. & Foster, J. L. (1974) Demonstration of hypoxic cell radiosensitization in solid tumours by metronidazole. *Br. J. Radiol.*, **47**, 399.

Brady, L. W. (Ed) (1980) *Radiation Sensitizers: Their Use in the Clinical Management of Cancer.* New York: Masson.

Clarke, C., Dawson, K. B., Sheldon, P. W., Chaplin, D. J., Stratford, I. J. & Adams, G. E. (1980) Quantitative cytochemical method for assessing the neurotoxicity of misonidazole. In *Radiation Sensitizers: Their Use in the Clinical Management of Cancer* (Ed. Brady). New York: Masson. p. 245.

Denekamp, J. (1979) Tumour-specific radiosensitizers for radiation therapy. *Radiobiol. Res. Radiother.*, **1**, 221.

DENEKAMP, J. & MICHAEL, B. D. (1972) Preferential sensitization of hypoxic cells to radiation *in vivo*. *Nature* (*New Biol.*), **239**, 21.

DENEKAMP J. MICHAEL, B. D. & HARRIS, S. R. (1974) Hypoxic cell radiosensitizers: Comparative tests of some electron affinic compounds using epidermal cell survival *in vivo*. *Radiat. Res.*, **60**, 119.

DISCHE, S., SAUNDERS, M. I. & STRATFORD, M. R. L. (1981) Neurotoxicity with desmethylmisonidazole. *Br. J. Radiol.*, **54**, 156.

FOSTER, J. L. & WILLSON, R. L. (1973) Radiosensitization of anoxic cells by Metronidazole. *Br. J. Radiol.*, **46**, 234.

MCNALLY, N. J., DENEKAMP, J., SHELDON, P. W. & FLOCKHART, I. R. (1978) Hypoxic cell sensitization by misonidazole *in vivo* and *in vitro*. *Br. J. Radiol.*, **51**, 317.

SMITHEN, C. E., CLARKE, E. D., DALE, J. A. & 4 others (1980) Novel (nitro-1-imidazolyl) alkanolamines as potential radiosensitizers with improved therapeutic properties. In *Radiation Sensitizers: Their Use in the Clinical Management of Cancer* (Ed. Brady). New York: Masson. p. 22.

WARDMAN, P. (1977) The use of nitroaromatic compounds as hypoxic cell radio-sensitizers. *Curr. Top. Radiat. Res. Quart.*, **II**, 347.

WATTS, M. E., ANDERSON, R. F., JACOBS, R. S. & 7 others (1980) Evaluation of novel hypoxic cell radiosensitizers *in vitro*. The value of studies in single cell systems. In *Radiation Sensitizers: Their Use in the Clinical Management of Cancer* (Ed. Brady). New York: Masson. p. 175.

WITHERS, H. R. (1967) The dose–survival relationship for irradiation of epithelial cells of mouse skin. *Br. J. Radiol.*, **40**, 187.